# Supplementary material for: COZOID: contact zone identifier for visual analysis of protein-protein interactions
Source: BMC Bioinformatics. 2018 Apr 6;19:125. doi: 10.1186/s12859-018-2113-6 (PMC5889581; doi:10.1186/s12859-018-2113-6)
Supplement: Supplementary file 2 — Software build. Executable binary file of the software tool. (ZIP 118,784 kb) [file 12859_2018_2113_MOESM2_ESM.zip › cozoid/platform/docs/testng-6.5.1-javadoc.zip!/org/testng/annotations/IFactoryAnnotation.html]

IFactoryAnnotation (TestNG)


---


|  |  |  |
| --- | --- | --- |
| **Overview** \| **Package** \| **Class** \| **Tree** \| **Deprecated** \| **Index** \| **Help** | |  |
| **PREV CLASS**   **NEXT CLASS** | **FRAMES**    **NO FRAMES**     **All Classes** |  |
| SUMMARY: NESTED \| FIELD \| CONSTR \| METHOD | DETAIL: FIELD \| CONSTR \| METHOD |  |


---


## org.testng.annotations Interface IFactoryAnnotation

**All Superinterfaces:**: IAnnotation, org.testng.internal.annotations.IDataProvidable, IParameterizable

---

``` public interface IFactoryAnnotation extends IParameterizable, org.testng.internal.annotations.IDataProvidable ```

Encapsulate the @Factory / @testng.factory annotation
Created on Dec 20, 2005

**Author:**
:   Cedric Beust

---

**Method Summary**

**Methods inherited from interface org.testng.annotations.IParameterizable**

`getEnabled, getParameters, setEnabled`

**Methods inherited from interface org.testng.internal.annotations.IDataProvidable**

`getDataProvider, getDataProviderClass, setDataProvider, setDataProviderClass`

---


|  |  |  |
| --- | --- | --- |
| **Overview** \| **Package** \| **Class** \| **Tree** \| **Deprecated** \| **Index** \| **Help** | |  |
| **PREV CLASS**   **NEXT CLASS** | **FRAMES**    **NO FRAMES**     **All Classes** |  |
| SUMMARY: NESTED \| FIELD \| CONSTR \| METHOD | DETAIL: FIELD \| CONSTR \| METHOD |  |


---
